# Paget's Osteosarcoma — no Cure in Sight

**DOI:** 10.1080/13577149977631

**Published:** 1999-12

**Authors:** Phillip J. Shaylor, David Peake, Robert J. Grimer, Simon R. Carter, Roger M. Tillman, David Spooner

**Affiliations:** The Royal Orthopaedic Hospital Oncology Service Bristol Road South Birmingham B 31 2AP UK

## Abstract

*Purpose* Paget' s osteosarcoma has a fearful reputation with a quoted survival of at best 5% at 5 years.We therefore reviewed our experience of 26 patients treated over the last 25 years using modern staging and limb salvage techniques to see if there had been any improvement in survival.

*Subjects:* We identified 26 patients on the Royal Orthopaedic Hospital Oncological database with a diagnosis of sarcoma secondary to Paget's disease.

*Results:* The survival rate was 53% at 1 year, 25% at 2 years and no patient survived for 5 years.The median survival was 21 months for those treated with curative intent and 7 months for those treated palliatively. Four of the five patients treated with limb-sparing surgery developed local recurrence between 5 and 12 months, the fifth died at 14 months.There was no difference in survival between amputation and limb salvage.

*Discussion:* The development of sarcomatous change in Paget's disease is well recognised. It represents an important segment of primary bone tumours in patients over 40 years of age.The prognosis is appalling. Indeed only 15 of 368 cases (4%) from a number of historical series have survived more than 5 years. Our results are similarly disappointing with no survivors at 5 years despite modern methods of management of bone tumours.While there is no difference in local recurrence rates or survival between limb reconstruction and limb ablation the poor prognosis for both means that neither can be recommended at present. Sarcomatous change in Pagetoid bone should therefore be regarded as a different disease to primary osteosarcoma. It remains an incurable disease with a poor prognosis.


Sarcoma (1999) 3, 191–192

ORIGINAL ARTICLE

### Paget's osteosarcoma — no cure in sight

PHILLIP J. SHAYLOR, DAVID PEAKE, ROBERT J. GRIMER, SIMON R. CARTER, ROGER M. TILLMAN & DAVID SPOONER

*The Royal Orthopaedic Hospital Oncology Service, Bristol Road South, Birmingham B31 2AP, UK*

**Abstract**

*Purpose* Paget's osteosarcoma has a fearful reputation with a quoted survival of at best 5% at 5 years. We therefore reviewed our experience of 26 patients treated over the last 25 years using modern staging and limb salvage techniques to see if there had been any improvement in survival.

*Subjects*: We identified 26 patients on the Royal Orthopaedic Hospital Oncological database with a diagnosis of sarcoma secondary to Paget's disease.

*Results*: The survival rate was 53% at 1 year, 25% at 2 years and no patient survived for 5 years. The median survival was 21 months for those treated with curative intent and 7 months for those treated palliatively. Four of the five patients treated with limb-sparing surgery developed local recurrence between 5 and 12 months, the fifth died at 14 months. There was no difference in survival between amputation and limb salvage.

*Discussion*: The development of sarcomatous change in Paget's disease is well recognised. It represents an important segment of primary bone tumours in patients over 40 years of age. The prognosis is appalling. Indeed only 15 of 368 cases (4%) from a number of historical series have survived more than 5 years. Our results are similarly disappointing with no survivors at 5 years despite modern methods of management of bone tumours. While there is no difference in local recurrence rates or survival between limb reconstruction and limb ablation the poor prognosis for both means that neither can be recommended at present. Sarcomatous change in Pagetoid bone should therefore be regarded as a different disease to primary osteosarcoma. It remains an incurable disease with a poor prognosis.

### Introduction

The development of sarcomatous change in Paget's disease of the bone is well recognised. Indeed five of the first 23 cases that were reported by Paget developed sarcoma.

The incidence of this complication is not known but has been reported to be between 0.7 and 15%[1–4] Previous reports have demonstrated the very poor prognosis of Paget's osteosarcoma with very few patients surviving 5 years.[5–10]

We therefore reviewed our experience of 26 patients treated over the last 25 years using modern staging and limb salvage surgery to see if there had been any improvement in the prognosis.

### Method

We identified 26 patients whom had been referred for advice or treatment on the Royal Orthopaedic Hospital Oncological database with a diagnosis of sarcoma secondary to Paget's disease. Eighteen patients were male and eight female. Their mean age was 70 years (range 58–89 years). Seventeen were stage 2b and 6 stage 3. The most common locations for the lesion were humerus and pelvis with eight cases each. There were seven cases involving the femur and one case involving each of the tibia, ulna and skull. Histologically 22 of the cases had classical Paget's osteosarcoma while the remaining four had a spindle cell sarcoma with no visible osteoid.

### Treatment

Eleven of the 26 patients underwent surgical resection with six having primary amputation and five having limb-sparing surgery with endoprosthetic replacement. In only 2 patients was it considered appropriate to use chemotherapy. Neither patient was considered to have benefited from chemotherapy. Five patients were treated with radiotherapy while 10 patients had palliative treatment only.

### Results

The survival rate was 53% at 1 year, 25% at 2 years and no patient survived for 5 years, all patients' dying of metastatic disease. The median survival was 21 months for those treated with curative intent and 7 months for those treated palliatively. Four of the five patients treated with limb sparing surgery developed

Correspondence to: Robert J. Grimer, The Royal Orthopaedic Hospital Oncology Service, Bristol Road South, Birmingham B31 2AP, UK.

1357-714X print/1369-1643 online/99/040191-02 © 1999 Taylor & Francis Ltd

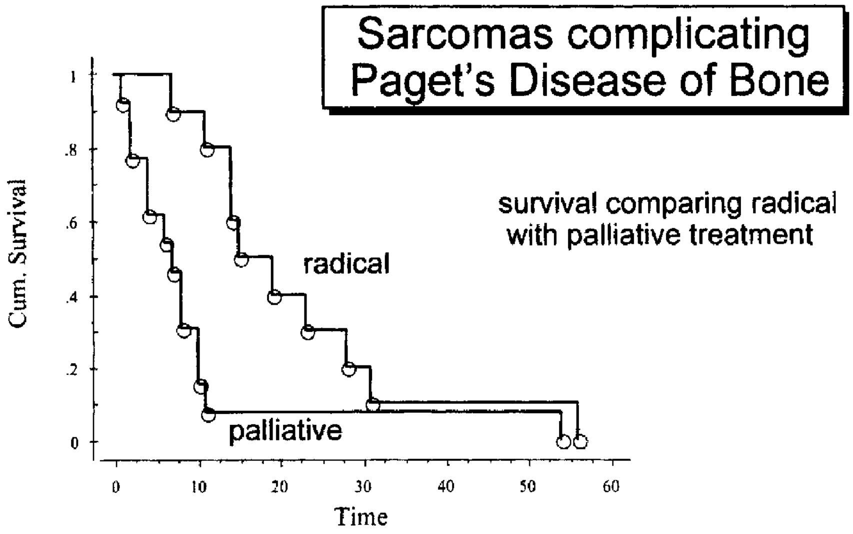

**Figure 1.** *Sarcomas complicating Paget's disease of bone.*

local recurrence between 5 and 12 months, the fifth died at 14 months. There was no difference in survival between amputation and limb salvage (Fig. 1).

### Discussion

The development of sarcomatous change in Paget's disease is well recognised. It represents an important segment of primary bone tumours in patients over 40 years of age. The prognosis is appalling. Indeed only 15 of 368 cases (4%) from a number of historical series have survived more than 5 years.

Our series is comparable to those of Greditzer, Scharjowicz and Smith[4–6] with a similar sex and age distribution. The site of disease is similarly mainly pelvis, femur and humerus. Histologically osteosarcomata are the most common type of sarcomatous change seen.

There have been many different approaches to treatment. Greditzer[6] performed ablative surgery in 18 cases with adjuvant chemotherapy and radiotherapy in six of theses but only had three survivors for 5 years. Huvos and Smith[4,7] have used adjuvant chemotherapy from 1974 but have a survival of six from 150 patients between their two series. Schajowicz[5] in his review of the Latin–American registry of Bone Tumours had only a 1.5% (one of 62) 5-year survival

Our results are similarly disappointing with no survivors at 5 years despite modern methods of management of bone tumours. There is no difference in local recurrence rates or survival between limb reconstruction and limb ablation and therefore neither can be considered curative. The decision as to which method of surgery should be offered for the individual patient will be based on which is felt to give the best palliation and the best function for that individual.

Sarcomatous change in Pagetoid bone should therefore be regarded as a different disease to primary osteosarcoma. It remains an incurable disease with a poor prognosis.

### References

1 Price CH. The incidence of osteogenic sarcoma in south-west England and it's relationship to Paget's disease of bone. *J Bone Joint Surg* 1962;44B:366–76.
2 Collins DH. Paget's disease of bone; incidence and subclinical forms. *Lancet* 1956;2:51–7.
3 Price CH, Goldie W. paget's sarcoma of bone; a study of 80 cases from the Bristol and Leeds Bone Tumour Registries. *J Bone Joint Surg* 1969;51B:205–24.
4 Smith J, Botet JF, The SDJ. bone sarcomas in Pagets disease: a study of 85 patients. *Radiology* 1984:152;583–90.
5 Schajowicz F, Arauto ES, Berenstein M. Sarcoma complicating Paget's disease of bone. A clinicopathological study of 62 cases. *J Bone Joint Surg* 1983;55B:299–307.
6 Greitzer HG, McLeod RA, Unni KK, Beabout JW. Bone sarcomas in Paget's disease. *Radiology* 1983;146:327–33.
7 Huvos AG, Butler A, Bretsky SS: Osteogenic sarcoma associated with Paget's disease of bone. *Cancer* 1983;52:1489–95.
8 Hadjipavlou A, Srolovitz H. Malignant transformation in Paget's disease of bone. *Cancer* 1992;70:2802–8.
9 Porretta CA, Dahlin DC, Janes JM. Sarcoma in Paget's disease of bone. *J Bone Joint Surg* 1957;39A:1314–28.
10 Barry HC. Sarcoma in Paget's disease of bone in Australia. *J Bone Joint Surg* 1961;43A:1122–34.
